# Antibacterial Properties of Non-Modified Wool, Determined and Discussed in Relation to ISO 20645:2004 Standard

**DOI:** 10.3390/molecules27061876

**Published:** 2022-03-14

**Authors:** Tomislav Ivankovic, Antonija Rajic, Sanja Ercegovic Razic, Sabine Rolland du Roscoat, Zenun Skenderi

**Affiliations:** 1 Department of Biology, Faculty of Science, University of Zagreb, 10000 Zagreb, Croatia; tona31@gmail.com; 2 Department of Materials, Fibres and Textile Testing, Faculty of Textile Technology, University of Zagreb, 10000 Zagreb, Croatia; sanja.ercegovic@ttf.unizg.hr (S.E.R.); zenun.skenderi@ttf.unizg.hr (Z.S.); 3 Laboratoire Sols, Solides, Structures et Risques (3SR), UMR 5521, Université Grenoble Alpes, CNRS G-INP, 38000 Grenoble, France; sabine.rolland-du-roscoat@univ-grenoble-alpes.fr

**Keywords:** textile, cotton, ISO standards, antimicrobial, agar diffusion

## Abstract

Wool is considered to possibly exhibit antibacterial properties due to the ability of wool clothing to reduce the build-up of odor, which arises from the microbial activity of skin microbiota. Indeed, when tested with a widely used agar diffusion plate test method, even wool or other textiles not treated with any antimicrobial agent can be interpreted to show certain antibacterial effects due to the lack of growth under the specimen, as instructed in ISO 20645:2004 standard. Therefore, we analyzed in detail what happens to bacterial cells in contact with untreated wool and cotton fabric placed on inoculated agar plates by counting viable cells attached to the specimens after 1 and 24 h of contact. All wool and several cotton samples showed no growth under the specimen. Nevertheless, it was shown without a doubt that neither textile material kills bacteria or inhibits cell multiplication. A reasonable explanation is that bacterial cells firmly attach to wool fibers forming a biofilm during multiplication. When the specimen was lifted off the nutrient agar surface, the cells in the form of biofilm remained attached to the wool fibers, removing the biomass and resulting in a clear, no growth zone underneath it. By imaging the textile specimens with X-ray microtomography, we concluded that the degree of attachment could be dependent on surface topography. The results indicate that certain textiles, in this case, wool, could exhibit antibacterial properties by removing excess bacteria that grow on the textile/skin interface when taken off the body.

## 1. Introduction

The possibility that wool has antibacterial properties comes from the ability of wool clothing to reduce/resist the onset of odor build-up, and odor is primarily considered to originate from microbial activity [1,2,3]. Caven et al. [1] suggested three possible explanations for the supposed antibacterial properties of wool. First, the complex wool fiber composed of epicuticle, lipid monolayer, and the cortex has an antibacterial effect, as suggested by Johnson et al. [3]. Nowadays, this statement does not seem to be correct, as several studies have undoubtedly shown that untreated wool itself does not exhibit bactericidal or bacteriostatic properties [1,4,5]. These studies used the absorption method (i.e., ISO 20743:2013) and compared the number of bacteria inoculated with liquid nutrient media onto the fiber specimen and the number of bacteria on the specimen after a certain period of time, showing that bacteria either remained viable or multiplied on the wool fibers. In one of our previous studies, the number of bacteria inoculated, according to the absorption method, on untreated wool fabric increased by four log values after 24 h of contact [6].

The second explanation would be that wool bonds or adsorbs odorous fumes without actually inhibiting bacterial growth, for which there is strong evidence [7,8,9].

The third possibility is that wool fibers’ hydrophobic surface and specific microstructure create a microclimate unfavorable for bacterial growth [1]. This third possibility was the object of our investigation. To simulate the real-life conditions where wool is in direct contact with skin covered with normal microbiota, we tested scoured plain weave woolen fabric by using the standard method ISO 20645:2004 “Textile fabrics—Determination of antibacterial activity—Agar diffusion plate test.” In this method, the textile specimen is placed on top of the inoculated agar, and as the antimicrobial agent diffuses into the agar, it either kills or stops bacterial cells from multiplying, giving a clear zone of “no bacterial growth” around the specimen. However, according to the method instructions, even the observation of no growth under the specimen can be classified as a “good effect” (the term “good effect” is a direct quote from the document).

However, there is a methodological issue with the agar diffusion test; the ISO 20645:2004 is suitable only to test the textiles treated with an antimicrobial agent compared with the control, untreated specimens [10,11]. However, earlier, we observed that untreated wool and some other untreated textiles also show “no growth” under the specimen when tested by the agar diffusion test [6]. Thus, can this be classified as an antibacterial effect?

**Figure 1:**
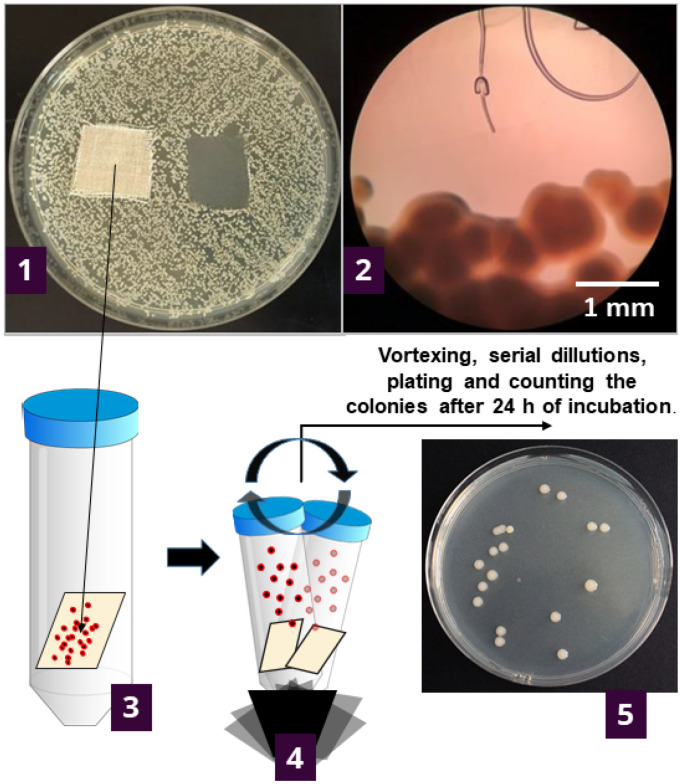
Experimental setup for determining the number of viable bacteria attached to the samples during the contact with inoculated agar. Initially, the bacteria are inoculated across the nutrient agar plate, and specimens are placed onto the plate. After the incubation (37 °C/24 h), the growth under the specimen was determined visually (**1**) and under the microscope (**2**). The textile specimen was transferred to 20 mL of sterile saline solution and vortexed five times for 5-sec bursts (**3**) to detach the bacterial cells from the specimen. The supernatant was serially diluted up to 10^–7^; plated and grown colonies were counted after the incubation at 37 °C/24 h (**4**). The number of bacteria was reported as log CFU·cm^−2^ of the specimen (**5**).

To try and provide an answer, we further investigated the results of agar diffusion testing by determining what is happening with the bacteria in contact with textile samples. This was undertaken by counting the viable cells on the specimens after 1 h and 24 h of contact of the textile specimen with agar inoculated with bacteria (Figure 1).

By doing this, we wanted to unravel the methodological issue of interpreting the “no growth” under the sample (instructed by ISO 20645:2004 method) and to define whether non-modified wool and other textiles adsorb the bacteria, kill the bacteria, or stop their growth, or do not have any effect on bacterial cells at all.

## 2. Results and Discussion

### 2.1. Agar Diffusion Test 

We assessed the antibacterial efficacy of three types of tested textiles, namely wool fabric, the cotton of a standard laboratory coat, and cotton of standard sterile compressed gauze, toward several bacterial species (Table 1). Bacterial species that were used in the present research were chosen to represent diversity regarding their metabolic and morphologic characteristics. The *Staphylococcus aureus* and *Klebsiella pneumoniae* are respectively Gram-positive and Gram-negative bacteria are listed as test organisms in ISO 20645:2004 standard. In our test battery, we added another Gram-positive bacterium, *Bacillus cereus*, which produces endospores. Finally, three strains of Gram-negative opportunistic pathogenic bacteria *Acinetobacter baumannii* were tested as well, an ATCC type strain and two hospital isolates resistant to multiple antibiotics.

**Figure 2:**
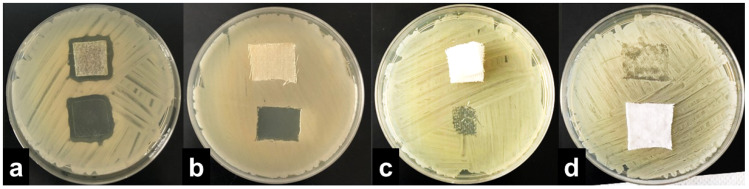
Interpretation of the agar diffusion test results based on ISO 20645:2004 standard: inhibition zone and no growth under the Aquacell^®^ specimen (**a**) in the experiment with *A. baumannii*. No inhibition zone and no growth under the wool fabric specimen (**b**) in the experiment with *B. cereus*. No inhibition zone and slight/moderate growth under the gauze specimen (**c**) in the experiment with *K. pneumoniae*. No inhibition zone and heavy growth under the cotton specimen (**d**) in the experiment with *S. aureus*.

The results were interpreted by adopting the ISO 20645:2004 assessment and observing the growth under the textile specimen (Table 1). “No growth” under the specimen (presented in Figure 2b) was assessed as good antibacterial effect, “slight growth” was assessed as a limit of efficacy whilst “moderate” (Figure 2c) or “heavy growth” (Figure 2d) was assessed as no effect. Aquacell^®^, a wound dressing doped with silver, was used as the positive control, showing no growth under the specimen along with an inhibition zone around the sample (Figure 2a), demonstrating antibacterial activity of ionic silver.

Comparing the three materials, the best antibacterial effect was exhibited by wool fabric (Table 1). Wool showed good antibacterial effect towards all tested bacteria except *S. aureus*, where it was at the limit of efficacy. Cotton was at the limit of efficacy towards all of the bacteria, again except *S. aureus* where it had insufficient effect, and two strains of *A. baumannii* where it showed good effect. Compressed gauze exhibited either insufficient effect or limit of efficacy. Comparing different bacteria, the results were similar for each material (Table 1), suggesting that the specific textile affects all bacteria in the same manner, again exempting *S. aureus*, which seemed to have the strongest growth under the specimen, regardless of the material. To summarise, out of the six different bacteria we tested, the wool exhibited good antibacterial effect toward five of them, the cotton toward two of them, and compressed gauze towards none (Table 1).

### 2.2. Comparison to Literature Data

The comparison of our results with literature data was somewhat challenging. A standardized method for antibacterial testing of textile samples resembling ours is perhaps the AATCC 147 Standard [12], where bacteria are inoculated on top of the agar plates as parallel streaks. The textile specimen is placed on the agar surface, and the inhibition zone around the specimen is monitored. Therefore, this method also enables direct contact of bacterial cells with the textile, assessment of growth under the sample, and can be used for textiles without diffusible agents [10]. We were able to find only one study reporting growth under the untreated wool sample; Liu et al. [13] tested capsaicin-coated wool fabric and reported “heavy growth” under control, untreated wool fabric, indicating no antibacterial effect. Another case of a very similar experimental setup was reported in Gomes et al. [14], where cotton modified with chitosan was tested by the JIS L 1902-Halo standard method. This method is very similar to ISO 20645:2004 and is also essentially a pour plate method; the difference is that “ISO” demands two layers of agar, the bottom layer clean and the upper layer inoculated with bacteria, while the “Halo method” demands only one layer of agar inoculated with bacterial culture. However, Gomes et al. [14] inoculated bacterial suspension on top of the agar layer, making it identical to our setup. They report no antibacterial effect on control untreated cotton, but only the “Halo zone”, the inhibition zone around the specimen, was monitored. There is no mention of bacterial growth under the specimen, preventing comparison to our results. A modified Kirby—Bauer test, a non-standardized method for antibacterial testing of textiles, is identical to the experimental setup we were using. However, we could not find any mention that growth under any textile, let alone wool, was monitored and reported, only the inhibition zone [15,16,17].

What is clear from the available literature is that wool itself does not exhibit bactericidal properties. Using the standard pour plate method, Pollini et al. [18] tested wool treated with silver and reported no antibacterial effect on untreated control samples. The same was reported in a couple of other studies [4,19]. From the aforementioned results, it is clear that the wool itself does not release any antibacterial agent whatsoever. However, as already mentioned, it also does not exhibit antibacterial activity when in contact with bacterial cells, as shown using the standard absorption method [1,4,5,6].

### 2.3. Viable Cell Count 

As we established that there was no bacterial growth under the majority of the wool specimens and most of the cotton specimens, and wool, according to literature, is not bactericidal itself. The question was what happened to the bacterial cells; were they inhibited to grow, attached to the textile and stopped multiplying or were they attached to the textile and multiplying? To answer this question, we determined the number of bacterial cells on the specimens after 1 h and 24 h of contact (specimens being placed onto an inoculated nutrient agar surface).

**Figure 3:**
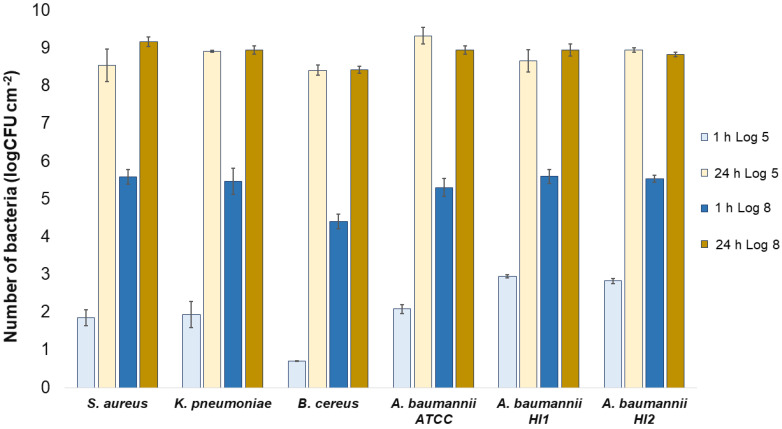
Numbers of viable bacteria that were adsorbed onto wool specimens after 1 h and 24 h of contact in the agar diffusion test. Two experimental setups, one in which the inoculum concentration was low (log 5 = the concentration was ~10^5^ CFU·mL^−1^) and the other in which the inoculum concentration was high (log 8 = the concentration was ~10^8^ CFU·mL^−1^). The inoculum was spread across the surface of the nutrient agar plates before the placement of specimens and incubation.

**Figure 4:**
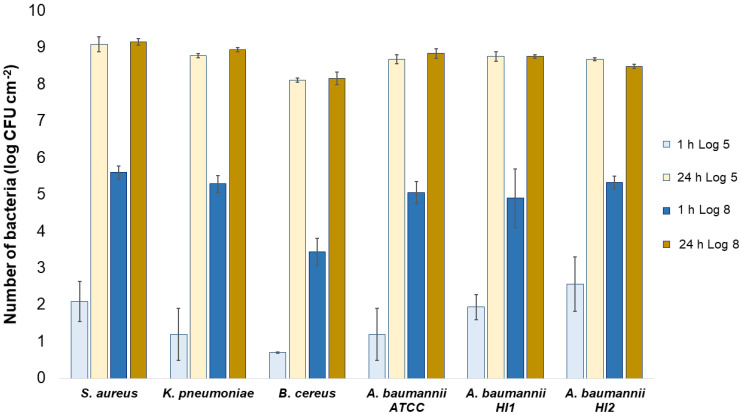
Numbers of viable bacteria that were adsorbed onto cotton specimens after 1 h and 24 h of contact in the agar diffusion test. Two experimental setups, one in which the inoculum concentration was low (log 5 = the concentration was ~10^5^ CFU·mL^−1^) and the other in which the inoculum concentration was high (log 8 = the concentration was ~10^8^ CFU·mL^−1^).

**Figure 5:**
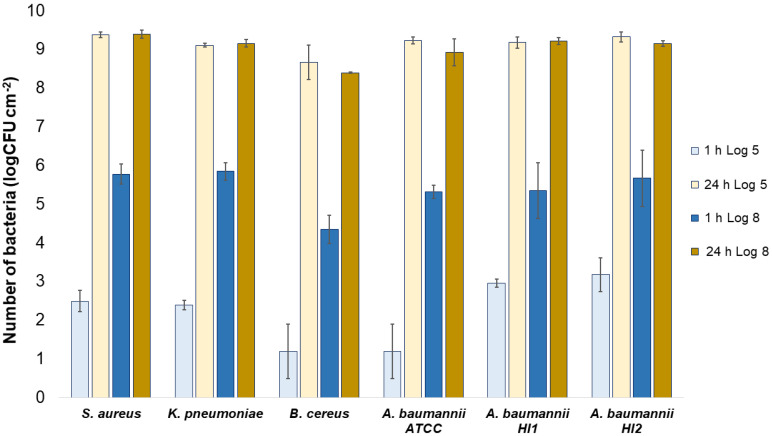
Numbers of viable bacteria that were adsorbed onto compressed gauze specimens after 1 h and 24 h of contact in the agar diffusion test. Two experimental setups, one in which the inoculum concentration was low (log 5 = the concentration was ~10^5^ CFU·mL^−1^) and the other in which the inoculum concentration was high (log 8 = the concentration was ~10^8^ CFU·mL^−1^).

All the bacterial species and all the textiles showed the same trend, meaning the bacteria attached to the materials during 1 h of contact and continued to multiply, as indicated by a significant (*p* < 0.05) increase in the bacterial numbers after 24 h of contact (Figure 3, Figure 4 and Figure 5). The same trend was in both experimental setups when the starting concentration of bacteria was lower (log 5 CFU·mL^−1^) or higher (log 8 CFU·mL^−1^). As expected, when initially fewer bacteria were inoculated on agar plates, the lower the number of bacteria attached after 1 h (Figure 3, Figure 4 and Figure 5). After 24 h the number of attached bacteria was the same, regardless of whether initially we inoculated a 1000-fold lower or higher concentration of bacteria (Figure 3, Figure 4 and Figure 5). Such results suggest that bacteria have initially attached and then multiplied until reaching some maximum amount, determined not by the number of bacteria initially attached to the textile but by the experimental setup (type of nutrient media, temperature, and time of incubation).

**Figure 6:**
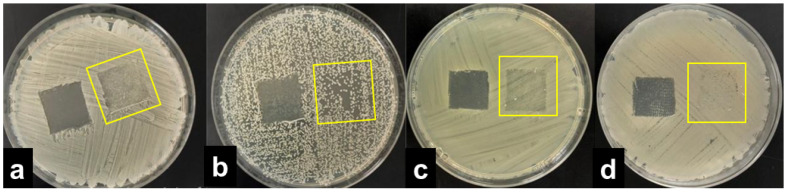
Results of the agar diffusion test in which the right specimen was removed after 1 h of contact and the left specimen was removed after 24 h of contact. (**a**,**b**)—experiments with wool and *S. aureus*; (**c**)—experiment with cotton and *A. baumannii*; (**d**)—experiment with compressed gauze and *B. cereus*.

Another question arose during the experiments, namely whether during 1 h of contact all of the bacteria that were inoculated on the surface of the agar plate attached to the textile specimen. The answer to this is no, as suggested by the fact that after removing the specimen after 1 h and incubating the agar plate, the bacteria continued to grow over the area where the specimen had been (Figure 6).

Now that we have established that none of the materials exhibited bactericidal or bacteriostatic activity, we can compare whether bacteria attach with a different affinity on different materials. 

**Figure 7:**
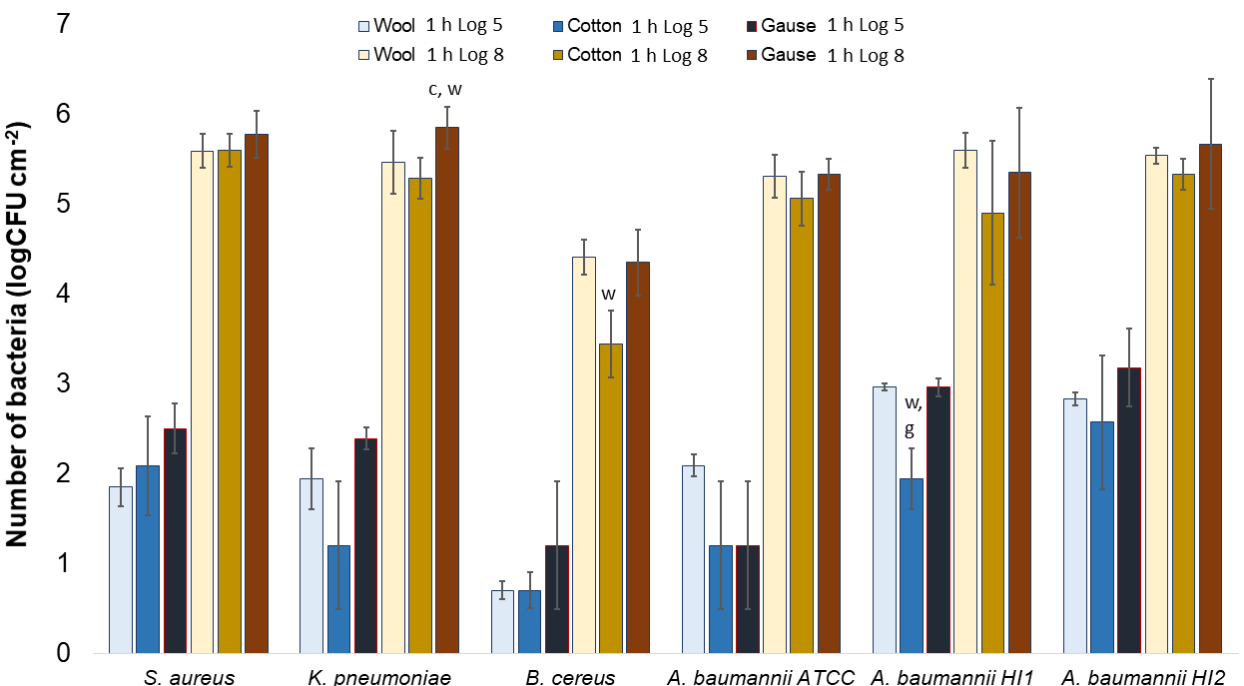
Numbers of viable bacteria that were adsorbed onto different specimens during the 1 h of contact in the agar diffusion test. Two experimental setups, one in which the inoculum concentration was low (log 5 = the concentration was ~10^5^ CFU·mL^−1^) and the other in which the inoculum concentration was high (log 8 = the concentration was ~10^8^ CFU·mL^−1^). w—statistically significantly different compared to wool; c—statistically significantly different compared to cotton; g—statistically significantly different compared to compressed gauze.

**Figure 8:**
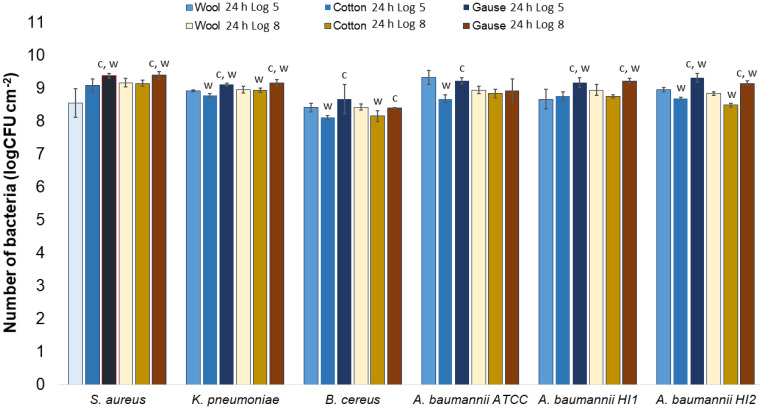
Numbers of viable bacteria that were adsorbed onto different specimens during the 24 h of contact in the agar diffusion test. Two experimental setups, one in which the inoculum concentration was low (log 5 = the concentration was ~10^5^ CFU mL^−1^) and the other in which the inoculum concentration was high (log 8 = the concentration was ~10^8^ CFU mL^−1^). w—statistically significantly different compared to wool; c—statistically significantly different compared to cotton; g—statistically significantly different compared to compressed gauze.

Generally, there was no notable difference in the number of bacteria attached to either wool, cotton, or gauze after 1 h of contact. Few statistically significant differences are marked in Figure 7, perhaps indicating a lesser affinity for bacterial adsorption by cotton when compared to wool or gauze. However, in any case, neither material has shown to clearly have a higher capacity for bacterial adsorption during 1 h of contact. On the contrary, after 24 h of contact, gauze had a statistically significantly higher number of bacteria when compared to wool or cotton in almost every experiment (Figure 8). The only exception is the experiment with *A. baumannii* ATCC19606 strain (Figure 8). Wool had a higher number of bacteria when compared to cotton in experiments with *K. pneumoniae*, *B. cereus*, and *A. baumannii* strains ATCC19606 and HI2 (Figure 8). By comparing the three materials, it would seem that all the materials initially adsorb the same amount of bacterial cells. However, during the 24 h of incubation, the bacterial growth is most intensive on the gauze than on cotton or wool. Yet, gauze was shown to have the strongest growth under the samples in the agar tests.

From all the observations, the possible explanation of antibacterial activity as interpreted by the agar diffusion method could be the following, when placed on the agar surface, the bacteria attach to the textiles and continue their growth, both on the textile fibres and the agar surface under the specimen. After 24 h of incubation, when textile specimens are lifted off the agar surface, the bacteria remain attached to the textile fibres, probably developing a biofilm. In the case of the wool fabric, all of the bacteria are firmly attached to the material, and almost none remain on the agar surface, while in the case of the gauze, many bacteria remain on the agar surface below the sample. It would seem that different structures and compositions of materials have different affinities for the development of bacterial biofilm. In addition, the affinity for biofilm development did not seem to depend on the bacterial species. The formation of bacterial biofilms on textile fibers is a well-known phenomenon. The affinity for biofilm development was linked to the textile’s hydrophobic and hygroscopic properties, surrounding environment, and bacterial species [20,21].

### 2.4. X-ray Microtomography

**Figure 9:**
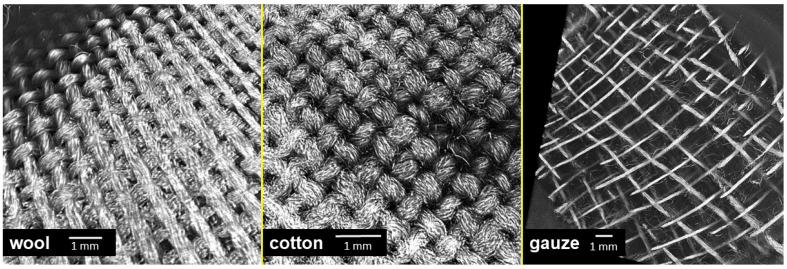
A 3D reconstruction of the surface of textile specimens used in the experiments, obtained by X-ray Microtomography.

In our experiments, the antibacterial effect of the tested textiles resulted from the strong attachment of bacterial cells on the samples, especially the wool fabric. Therefore, we examined the surface topography of the tested materials by using X-ray Microtomography (XT). This method allows visualization of textile surfaces that were in contact with the agar surface on the mesoscale and not just on the microscale (i.e., few fibres) as with electron microscopy. In addition, the wool and cotton fabrics had a similar topography (Figure 9), while compress gauze differed significantly (Figure 9). The images could explain the apparent heavy growth under the gauze samples; the gauze material had a higher porosity than the other samples. Only a small part of the fibres are in direct contact with the agar surface, while wool and cotton were in contact with agar with most of their surface. The bacteria probably developed a strong biofilm only on the fibres in direct contact with the agar and thus were removed from the surface in the tests with wool and cotton and remained on the agar surface in the tests with the compress gauze leaving significant biomass under the gauze samples.

## 3. Materials and Methods

### 3.1. Bacterial Strains

The experiments were performed using the *Staphylococcus aureus* (ATCC 25 923), *Klebsiella pneumoniae* (ATCC 11296), *Bacillus cereus* (LBK 4080), and *Acinetobacter baumanii* ATCC 19606 strain along with two multiple drug resistant hospital isolates, marked as HI1 and HI2. All the bacteria were kept cryo-stored using the MicrobankTM system (Pro-Lab diagnostics, Richmond Hill, ON, Canada). The bacteria were grown on Tryptic soy agar (TSA) plates (37 °C/24 h) prior to the start of the experiments.

### 3.2. Textile Materials

To test the antibacterial properties of untreated textiles, one sample of scoured wool fabric and two cotton samples were used in the experiment. The plain weave woolen fabric of linear density (for warp/weft) 15.5 × 2/25 tex, count (warp/weft) of 21/24 cm^−1^, and mass per unit area of 140 g/m^2^, is labeled as (W) and was industrially prepared (washed, decatised, sheared and dried) and supplied by Varteks Ltd. (Varazdin, Croatia). For comparison, two samples of cotton material of different degrees of finishing were also tested. The first cotton sample was a standard laboratory white coat (C) in twill with an embroidery count (warp/weft) of 18/17 cm^−1^ and mass per unit area of 220 g/m^2^, manufactured by Marija Ltd. (Zagreb, Croatia). The second one was a standard sterile compressed gauze (G) in canvas embroidery, with a count (warp/weft) of 11/8 cm^−1^ and mass per unit area of 40 g/m^2^, manufactured by Lianyungang Ruikang Sanitary Dressing Company Ltd. (Lianyungang, China). For a positive control, a textile with known antibacterial properties, Aquacell^®^ wound dressing that is incorporated with ionic silver (Convatec Inc., Berkshire, UK). The textiles were cut into 20 × 20 mm squares and used in the experiment without prior sterilization, as noted in ISO 20645:2004 standard method. However, disinfected nitrile hand gloves were used during the cutting to minimize contamination of samples from the skin bacteria.

### 3.3. Antibacterial Testing

The procedure of antibacterial testing (Figure 1) was based on a method described in ISO 20645:2004—“Textile fabrics—Determination of antibacterial activity—Agar diffusion plate test” (reference ISO 2004). Suspensions of concentration 10^5^ and 10^8^ Colony Forming Units (CFU) per mL of sterile 0.3% saline were made for each tested bacterium. Such solutions were made by dispersing bacterial biomass in sterile saline up to 0.5 McFarland units (corresponding to ~10^8^ cells per mL). Next, the solution was serially diluted to obtain 10^5^ CFU mL^−1^ suspension. The CFU’s were checked by plating prior to each experimental batch. To grow the “bacterial lawn”, bacterial suspension was spread across the Tryptic soy agar (Biolife, Italy) plate using a sterile cotton swab. After plate inoculation, two textile specimens (20 × 20 mm) were placed on the agar surface using sterile tweezers. The agar plates were then incubated for 24 h at 37 °C. The results were interpreted according to ISO 20645:2004 standard by examining colony growth under the textile sample, visually and under the microscope (Olympus Japan, CX21) at 40× magnification (Figure 1). 

### 3.4. Determining the Number of Bacteria Attached to Textile Samples

To determine if and in what amount the bacteria remain attached to textiles during the antibacterial testing (as previously described), the samples were gently removed (Figure 1) and immersed in 20 mL of sterile saline (in 50 mL Falcon-type tubes). The tubes were shaken-out on a vortex shaker according to ISO 20743:2013—“Textiles—Determination of the antibacterial activity of textile products”, for 5 × 5 s cycles. Shaking detaches the bacteria from the fabric, and the cells remain free-floating in the saline suspension. A total of 1 mL of suspension was serially diluted up to 10^−7^, and 0.1 mL was inoculated on TSA plates. After the incubation (24 h/37 °C) the grown colonies were counted, and the bacterial numbers were reported as CFU per cm^2^ of textile material. As a control, clean textile specimens were used, meaning they were not previously incubated on agar plates. Several bacterial colonies usually grew as the specimens were not sterile, but total counts were less than 10 CFU·mL^−1^. 

### 3.5. X-ray Microtomography

X-ray microtomography was performed to visualize the inner structure of the three types of textiles at the micron scale. The 3D images of the textiles were obtained on a laboratory tomograph manufactured by RX Solutions (Annecy, France) equipped with a Hamamatsu X-ray source (Hamamatsu City, Japan) and a Varian flat panel detector (Varian Medical Systems, Salt Lake City, UT, USA). Each sample was irradiated with an X-ray beam (generated with a 100 kV 100 μA electron beam on a tungsten target) for 2400 angular projections equally spaced over 360°. The 2D radiographs were converted into a 3D dataset using a filtered back projection algorithm, and 3D views were obtained using the 3D viewer of Fiji software (v 1.52f). The chosen pixel size was set to 10 μm. This pixel size was chosen as it allows the visualization of the fibers and the fiber bundle that constitute the textile and a representative volume of the textile as many periods of the structure can be seen.

### 3.6. Statistical Analysis

All the experiments were undertaken in triplicate. The growth under the textile specimen was determined qualitatively by visual inspection. The numbers of bacteria attached to textile samples were quantitatively compared and analyzed using Statistica^®^ software (StatSoft, Tulsa, OK, USA). Ordinary Student’s t-test was used, and statistical significance was set at *p* < 0.05.

## 4. Conclusions

The wool fabric showed antibacterial efficacy towards several bacterial species if interpreted according to the agar diffusion test as “no growth” under the textile sample. On the other hand, experiments monitoring the number of viable cells after 24 h of bacteria/textile contact showed that neither the wool sample nor two different cotton samples exhibited any bactericidal or bacteriostatic activity in terms of inactivating or killing bacterial cells. Instead, bacterial cells readily multiplied on the textile samples during 24 h of incubation on nutrient agar plates. The explanation would thus be that bacteria strongly adsorb to wool while actively multiplying, developing a firmly attached biofilm. When the wool sample was lifted off, the bacterial biofilm remained attached to wool fibers, removing the biomass from the surface of the nutrient agar, resulting in a clear “no growth” zone under the sample.

Since similar observations were present in experiments with cotton but not with compress gauze, it would seem that the surface topography and structure of the textile plays an important role in the antibacterial efficacy of the textiles which are unmodified with some antibacterial agent. The results indicate that certain textiles, in our case woven wool fabric, could exhibit antibacterial properties by removing excess bacteria that grow on the textile/skin interface when taken off the body.

A deeper analysis of biofilm formed on the textile fibers, visualized by scanning electronic microscopy, and quantified as it develops, would also be desirable. Along with further tests with different non-modified textile materials and the same materials of differing structure and porosity, the question of why some textiles seem to show antibacterial efficacy without showing any bactericidal activity could be resolved.

## Figures and Tables

**Table 1:** Assessment of antibacterial efficacy of textile materials determined with the ISO 20645:2004 “Agar diffusion plate test”. The material/bacteria combination that yielded “good effect” is shaded.

Bacteria	Sample	Growth Under the Specimen	Assessment of Antibacterial Efficacy Adopted from ISO 20645:2004
*S. aureus* ATCC 25923	Wool	none/slight	good effect/limit of efficacy
	Cotton	heavy	insufficient effect
	Gauze	slight/moderate	limit of efficacy/insufficient effect
*K. pneumoniae* ATCC 11296	Wool	none	good effect
	Cotton	none/slight	good effect/limit of efficacy
	Gauze	slight	limit of efficacy
*B. cereus* LBK 4080	Wool	none	good effect
	Cotton	none/slight	good effect/limit of efficacy
	Gauze	slight/moderate	limit of efficacy/insufficient effect
*A. baumannii* ATCC 11296	Wool	none	good effect
	Cotton	none	good effect
	Gauze	slight	limit of efficacy
*A. baumannii* HI1	Wool	none	good effect
	Cotton	none/slight	good effect/limit of efficacy
	Gauze	slight	limit of efficacy
*A. baumannii* HI2	Wool	none	good effect
	Cotton	none	good effect
	Gauze	slight	limit of efficacy

## Data Availability

Not applicable.

## Sample Availability

Samples of the compounds are available from the authors.
